# Systematic identification of chicken type I, II and III interferon-stimulated genes

**DOI:** 10.1186/s13567-020-00793-x

**Published:** 2020-05-24

**Authors:** Manman Dai, Tingting Xie, Ming Liao, Xiquan Zhang, Min Feng

**Affiliations:** 1 Guangdong Provincial Key Laboratory of Agro-animal Genomics and Molecular Breeding, College of Animal Science, South China Agricultural University, Guangzhou, China; 2 College of Veterinary Medicine, South China Agricultural University, Guangzhou, China; 3 Key Lab of Chicken Genetics, Breeding and Reproduction, Ministry of Agriculture, Guangzhou, Guangdong China

## Abstract

Interferon-stimulated genes (ISGs) play an important role in antiviral innate immune responses. Although many ISGs have been identified in mammals, researchers commonly recognize that many more ISGs are yet to be discovered. Current information is still very limited particularly for the systematic identification of type III ISGs. Similarly, current research on ISGs in birds is still in its infancy. The aim of this study was to systematically identify chicken type I (IFN-α), II (IFN-γ) and III (IFN-λ) ISGs and analyze their respective response elements. RNA sequencing (RNA-Seq) was employed to identify those genes with up-regulated expression following chicken IFN-α, IFN-γ and IFN-λ treatment. Two hundred and five type I ISGs, 299 type II ISGs, and 421 type III ISGs were identified in the chicken. We further searched for IFN-stimulated response elements (ISRE) and gamma-activated sequences (GAS) elements in the promoters region of ISGs. The GAS elements were common in the promoter of type II ISGs and were even detected in type I and III ISGs. However, ISRE were not commonly found in the promoters of chicken ISGs. Furthermore, we demonstrated that ISRE in chicken cells were significantly activated by IFN-α or IFN-λ treatment, and expectedly, that GAS elements were also significantly activated by IFN-γ treatment. Interestingly, we also found that GAS elements were significantly activated by IFN-λ. Our study provides a systematic library of ISGs in the chicken together with preliminary information about the transcriptional regulation of the identified ISGs.

## Introduction

Based on sequence homology and receptor specificity, interferons (IFNs) are divided into three types, i.e. type I, II and III [1]. The three types of IFNs display distinct expression patterns and have each key role in innate and adaptive immunity. Interestingly, the first identified IFN was chicken interferon, originally defined as a factor that interferes with influenza virus replication in chicken chorioallantoic membrane [2]. However, after its initial discovery, the IFN related research in chicken immunology has been lagging behind, especially in the field of antiviral mechanisms and its application to combat viral disease in chicken. Chicken IFNs (ChIFNs) also include the three types present in mammals. The difference is that the identified type I ChIFNs include both IFN-α and IFN-β, whereas type II (IFN-γ) and type III (IFN-λ) ChIFNs exist as a single gene each [3]. In fact, as a broad-spectrum antiviral agent, IFNs do not directly kill or inhibit the virus but rather act indirectly as autocrine or paracrine factors to activate the JAK-STAT signaling pathway and transcriptionally induce a large number of Interferon-stimulated genes (ISGs) that exert the antiviral effects [1, 4].

ISGs, which are important in the control of viral infections, can be induced by IFNs, viruses, IFN regulatory factor 1 (IRF1) and IRF7, among other substances [4–6]. In the canonical pathway for generating ISGs, IFN-induced signaling initially results in the phosphorylation of receptor-associated tyrosine kinases of the Janus kinase (JAK) family of proteins, which then phosphorylate the signal transducer and activator of transcription (STAT) family of proteins (STAT1 and STAT2). Phosphorylated STAT1 and STAT2 (during type I or III IFN stimulation) form a heterodimer that translocates to the nucleus to form the heterotrimeric transcription factor complex consisting of IFN-stimulated genes factor 3 (ISGF3) with IRF-9, thereby inducing hundreds of ISGs after binding the IFN-stimulated response elements (ISREs). However, activation by type II IFN only involves the dimerization of phosphorylated STAT1 to form γ-interferon activation factor (GAF) that translocates to the nucleus and binds to gamma-activated sequences (GAS) of response genes [7].

It is well documented that the human library of ISGs has been steadily expanding from 1998 to 2018 through the application of high throughput screening methods [8–12]. These previously identified-ISGs are induced by IFN-α, β, or γ in various cell types. Unfortunately, information on ISGs induced by type III interferon (IFN-λ) is very limited. Recently, studies have shown that IFN-λ plays an important role in antiviral defense in epithelia [13], which underscores the importance of ISGs induced by IFN-λ.

The zoonotic avian influenza viruses, including H7N9, H10N8, H5N1, and H5N6 have the potential to cause serious disease with alarmingly high fatality rates among humans and are considered a major public health threat because of their potential to trigger of pandemic influenza outbreaks of avian origin [14, 15]. Both IFN-α and IFN-λ play an important role in combating influenza viruses [16–18]. Furthermore, IFN-λ is considered more effective than type I IFNs in protecting against influenza virus [16]. Both chicken types I and II IFNs appear to protect fibroblasts from viral infection [19–22]. However, chicken type III IFN acts predominantly on epithelial cells [23]. The antiviral effect exerted by IFNs is achieved by inducing the production of ISGs [4]. Therefore, a comprehensive catalog of ISGs in poultry could be useful information for the prevention and control of zoonotic avian influenza viruses.

Compared to mammals, limited information is presently available on avian ISGs. IFN-α-induced ISGs have been identified in different cell types of chicken, according to our and other studies [24, 25]. Recently, chicken ISGs induced by type I, II and III IFN were also identified in fibroblasts model at 24 h post IFN treatment [26]. Although hundreds of ISGs have been identified in humans, several more ISGs are expected to be still discovered [12]. During the identification of ISGs, different cell types, different time points of IFN stimulation, and different methods may yield different results [4]. As the latest version of the chicken genome (Gallus_gallus-6.0) is available, we decided to also update the identification of chicken ISGs.

In the present study, we systematically identified chicken ISGs induced by type I, II, and III IFNs, and further analyzed the interferon response elements in the promoters of the identified chicken ISGs.

## Materials and methods

### Cell culture

HEK293E cells (Sino Biological Inc., Beijing, China) were used to transiently express the interferon protein and were cultured in SMM 293-TI medium (Sino Biological Inc.) at 37 °C under 5% CO_2_. A continuous cell line of chicken embryo fibroblasts, DF1 cells, [27], were maintained in Dulbecco’s modified Eagle medium (DMEM) (Gibco, California, USA) supplemented with 10% fetal bovine serum (Gibco). The first established domestic fowl epithelial cell line, LMH [28] was maintained in DMEM/F-12 (Gibco) supplemented with 10% fetal bovine serum. DF1 and LMH cells were purchased from ATCC (Manassas, VA, USA) and kept in Guangdong Provincial Key Laboratory of Agro-animal Genomics and Molecular Breeding.

### Interferons

Chicken interferon-α (ChIFN-α) was purchased from GenWay Biotech (San Diego, USA). ChIFN-γ and ChIFN-λ were obtained via eukaryotic expression using HEK293E cells. Briefly, the coding DNA sequences for ChIFN-γ (NM_205149.1) and ChIFN-λ (NM_001128496.1) were synthesized (Genecreate, Wuhan, China) and were respectively cloned into the pCMV3-c-His-vector (Sino Biological Inc.). The pCMV3-ChIFN-γ and pCMV3-ChIFN-λ plasmids were transfected into HEK293E cells using the Sinofection Transfection Reagent (Sino Biological Inc.). Transfected cells were maintained in SMM 293-TI medium (Sino Biological Inc.) supplemented with SMS 293-SUPI (Sino Biological Inc.) at 37 °C under 5% CO_2_ at 175 rpm. Transfected HEK293E cells were cultured for 7 days before harvesting. ChIFN-γ and ChIFN-λ proteins were purified using immobilized metal affinity chromatography. Protein concentrations were determined by the bicinchoninic acid (BCA) protein assay (Beyotime, Shanhai, China). The purified ChIFN-γ and ChIFN-λ proteins were analyzed via sodium dodecyl sulfate polyacrylamide gel electrophoresis (SDS-PAGE) and Western blot using His antibodies and ChIFN-γ and ChIFN-λ serum polyclonal antibodies.

The activity of ChIFN-γ and ChIFN-λ proteins was analyzed by inhibiting the vesicular stomatitis virus (VSV)-induced cytopathic effects (CPE) on DF1 cells and LMH cells (ChIFN-λ), as previously described [29]. In brief, DF1 or LMH cells were seeded in 96-well plates and were treated with 100 μL fourfold diluted ChIFN with the starting dilution of 1:4. After 12 h, media containing the recombinant ChIFN was removed and the cells were washed with PBS. Attached cells were then infected with 100 TCID_50_ VSV for 24 h. The CPE was observed under microscope and the number of CPE wells was statistical. Cell wells containing ChIFN without VSV were used as the negative controls. Cells treated by VSV but lacking ChIFN were used as positive controls. Assay results were calculated according to the method of Reed &Muench and expressed as UI/mg.

### Library preparation for mRNA sequencing

After growing into a single layer in a 12-well cell culture plate, DF1 cells were treated with ChIFN-α, ChIFN-γ and ChIFN-λ (1000 UI/mL), respectively. Given that many studies have found that IFN-λ mainly plays a role in epithelial cells [30, 31], LMH, the first established domestic fowl epithelial cell line [28], was also used to identify ISGs induced by ChIFN-λ. ChIFN-α, ChIFN-γ, and ChIFN-λ proteins were added to culture media, respectively, to a final concentration of 1000 UI/mL in DF1 cells. The LMH cells were only treated with ChIFN-λ (1000 UI/mL). DF1 and LMH cells were treated with ChIFN or phosphate-buffered saline (PBS) (natural control, NC) and incubated for 6 h before being harvested. Total RNA was isolated from these cells for RNA-Seq, using the TRIzol reagent (Invitrogen, CA, USA). Samples were collected from two independent experiments.

Approximately 3 μg of RNA per sample was used as input material. The mRNA was enriched by Oligo(dT) beads and then split into short fragments using fragmentation buffer. The fragments were then reverse transcribed into cDNA using random primers. Second-strand cDNA was synthesized by DNA polymerase I, RNase H, and dNTP. The cDNA fragments were then purified using the QiaQuick PCR extraction kit, poly(A) tails were added, and the ends were repaired and ligated to Illumina sequencing adapters. The ligation products were size selected by agarose gel electrophoresis, PCR amplified, and were then sequenced using the Illumina HiSeqTM 2500 by Gene Denovo Biotechnology Co. (Guangzhou, China). The sequencing data were deposited in the Bioproject database under the Bioproject IDs: PRJNA539825 and PRJNA539821.

### RNA-Seq data analysis

To acquire high quality clean reads, the raw reads were filtered by removing the adapter-containing reads, low quality bases including reads with more than 10% unknown nucleotides, and low-quality reads with more than 50% of low-quality bases (Q-value ≤ 20). Bowtie2, a short reads alignment tool, was used to remove ribosomal RNA (rRNA) [32]. The remaining clean reads were mapped to the latest version of the chicken genome assembly (Gallus_gallus-6.0) using TopHat2 (version 2.0.3.12) [33]. The mapped reads of each sample were assembled using the Cufflinks and Cuffmerge software. Gene abundances were then quantified using the RSEM software [34] and gene expression level was normalized using FPKM (Fragments Per Kilobase of transcript per Million mapped reads).

The edgeR package [35] was used to identify differentially expressed genes (DEGs) across groups (ChIFN vs NC). Genes with a fold change (FC) of |log_2_FC| > 1 and a false discovery rate (FDR) < 0.05 were considered DEGs.

Gene ontology (GO) enrichment analysis provides all GO terms that are significantly enriched in DEGs compared to the genome background, and filter the DEGs that correspond to biological functions. All DEGs were mapped to GO terms in the Gene Ontology database [36], gene numbers were calculated for every term, and significantly enriched GO terms in DEGs compared to the genome background were identified by hypergeometric test. GO has three ontologies including molecular function, cellular component and biological process. For the pathway enrichment analysis, the DEGs were mapped to Kyoto Encyclopedia of Genes and Genomes (KEGG) database.

In the present study, the up-regulated DEGs were identified as chicken ISGs. IGSs induced by different types of ChIFN were also subjected to GO and KEGG analysis.

### Quantitative reverse transcription-PCR

To verify chicken ISG expression, cDNA synthesis was performed using the PrimeScript RT Reagent Kit (TaKaRa, Dalian, China) according to the manufacturer’s protocol. Furthermore, qPCR analysis was performed on a Bio-Rad CFX96 Real-Time Detection System using iTaq™ Universal SYBR^®^ Green Supermix Kit reagents (Bio-Rad, CA, USA) according to the manufacturer’s specifications. The specific qPCR primers were designed using the National Center for Biotechnology Information (NCBI) Primer BLAST program. The *GAPDH* gene was used as an internal control. qPCR results are representative of three independent experiments. Data analyses were performed using the 2^–ΔΔCt^ method [37].

### Analysis of ISRE and GAS elements

To determine whether the identified type I, II, and III chicken ISGs contained ISRE and GAS elements in their promoter regions, we searched for these elements in the respective genes, according to the methods outlined in a previous report [38]. Based on previous work [38, 39], the 6-kb sequence upstream of the first exon was considered the gene promoter region. Therefore, the sequences 6 kb upstream of the first exon of the chicken ISGs identified in the present study were individually searched for the existence of the common ISRE, 5′ A/GGTTTCN_(1-2)_TTTCC/T 3′ or its reverse complement and the common GAS, 5′ TTNCNNNAA′ [38, 40, 41].

### Luciferase reporter assays

Plasmid pGL3-chicken 4× MxISRE (MxISRE); pGL3-chicken ISRE (chISRE); and pGL3-chicken GAS (chGAS) were constructed in this study. Briefly, plasmid pGL3-MxISRE was constructed by inserting 4× known chicken Mx ISRE (AGTTTCGTTTCT) [39] into the pGL3-Basic vector (Promega, Madison, USA). Plasmid pGL3-chISRE and pGL3-chGAS were constructed via gene synthesis by concatenating many functional ISRE and GAS elements, according to the methods described in previous studies [38, 42].

Luciferase reporter assays were performed on monolayers of DF1 or LMH cells in 48-well plates. DF1 and LMH cells were co-transfected with pGL3-MxISRE and pRL-TK; or pGL3-chISRE and pRL-TK; or pGL3-chGAS and pRL-TK using Lipofectamine 3000 (Invitrogen), according to the manufacturer’s protocol. Cells were co-transfected with pGL3-basic and pRL-TK as the control. At 24 h post-transfection, the transfected DF1 cells were respectively treated with ChIFN-α, ChIFN-γ, and ChIFN-λ. In contrast, the transfected LMH cells were treated with ChIFN-λ alone. Measurements of reporter luciferase activity were carried out at 6 h post IFN treatment, using the Dual-Luciferase Reporter Assay System, according to the manufacturer’s directions (Promega). Luminescence was measured using a Fluorescence/Multi-Detection Microplate Reader (BioTek, Winooski, USA). Firefly luciferase activities were normalized to Renilla luciferase luminescence in each well. For each group, transfections were done in triplicate. Assays were performed three times.

### Statistical analyses

Statistical comparisons were performed using GraphPad Prism 5 (GraphPad Software Inc., San Diego, CA, USA). The results were presented as the mean ± SEM. Statistical significance was set at *P* values of > 0.05 (non-significant, ns), < 0.05 (*), 0.01 (**) or 0.001(***).

## Results

### Preparation of ChIFN-γ and ChIFN-λ

**Figure 1:**
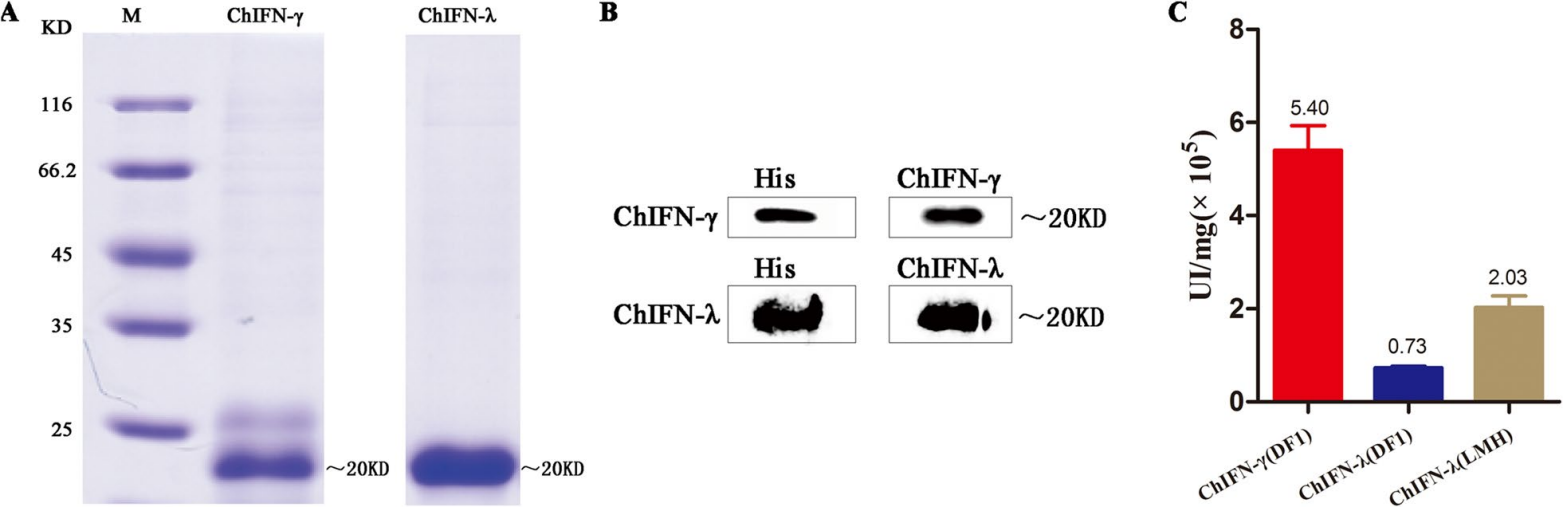
**Preparation of chicken types II and III interferon. A** Chicken IFN-γ and IFN-λ (ChIFN-γ and ChIFN-λ) were prepared after expression in HEK293E cells. Purified ChIFN-γ and ChIFN-λ samples were subjected to SDS-PAGE. **B** The purified proteins were further verified by Western blotting using His antibody and ChIFN-γ and ChIFN-λ serum polyclonal antibody. **C** The bioactivity of the purified ChIFN-γ and ChIFN-λ proteins was detected in DF1 and LMH cell lines. The results are representative of three independent experiments.

ChIFN-γ and ChIFN-λ were prepared after over-expression in HEK293E cells. Purified samples were subjected to SDS-PAGE which showed recombinant ChIFN-γ and ChIFN-λ proteins (Figure 1A). The purified proteins were further verified by Western blotting using His antibodies, and ChIFN-γ and ChIFN-λ serum polyclonal antibodies (Figure 1B). We prepared 1 mg of pure recombinant ChIFN-γ and ChIFN-λ. Furthermore, after anti-VSV activity analysis, the bioactivity of the purified ChIFN-γ protein in DF1 cells was calculated at 5.4 × 10^5^ UI/mg (IFN activity units per mg) using the Reed-Muench method (Figure 1C). The bioactivity of the purified ChIFN-λ protein in DF1 and LMH cells was calculated as 7.34 × 10^4^ UI/mg and 2.0 × 10^5^ UI/mg, respectively (Figure 1C). The inhibition of cytopathic effects induced by VSV (100 TCID_50_) under different dilutions of IFN is shown in Additional file 1.

### Analysis of DEGs by RNA sequencing

RNA-Seq was employed to identify genes that are induced by chicken type I, II and III IFN, respectively. Eight cDNA libraries were constructed using the total RNA of DF1 cells from two control samples and six IFN treatment samples. Four cDNA libraries derived from LMH cells were constructed from two control samples and two ChIFN-λ treatment samples (Additional file 2). The Illumina HiSeq 2500 platform produced 75,549,654,300 bp of raw data and 488,264,396 clean reads (Additional file 2). The clean reads were mapped onto the chicken reference genome (Gallus_gallus-6.0), and the mapping rate of each library ranged from 84.45% to 87.28% (Additional file 2).

Based on the threshold of fold change (FC) of |log_2_FC| > 1 and a false discovery rate (FDR) < 0.05, genes in different types of ChIFN-treated groups were classified into 3 categories including up-regulated DEGs, down-regulated DEGs and equally- regulated genes and displayed in volcano plots (Additional file 3).

**Figure 2:**
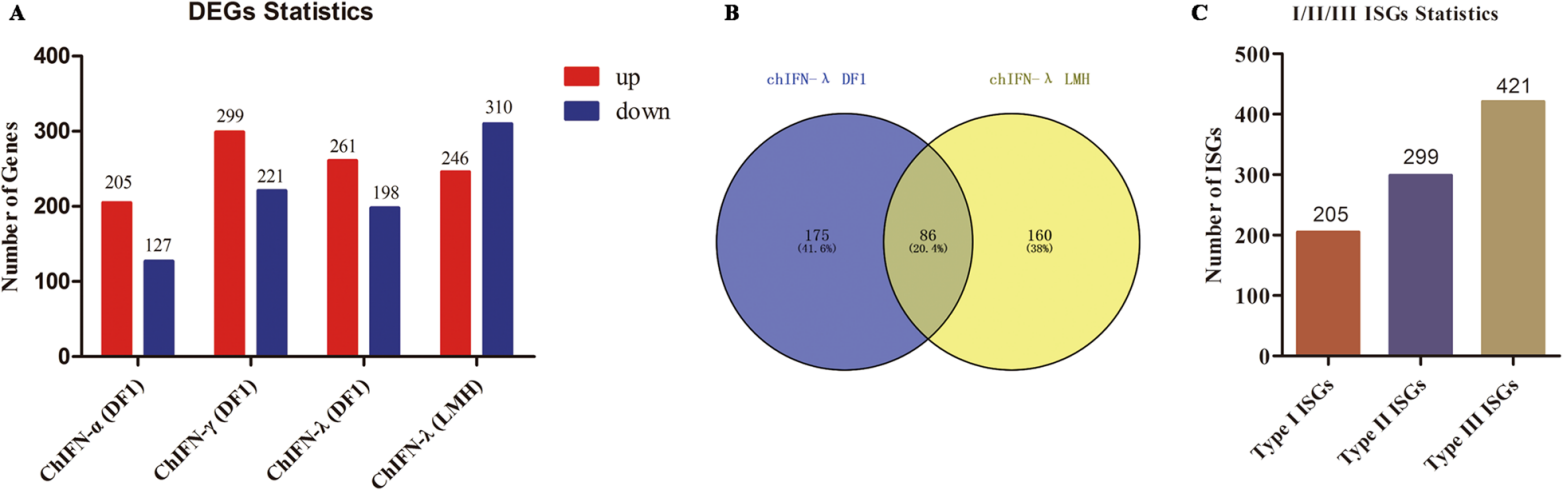
**Systematic identification of chicken type I, II and III ISGs. A** Differentially expressed genes (DEGs) in chicken IFN-stimulated cells as detected by RNA-Seq. **B** Venn diagrams of up-regulated DEGs in DF1 and LMH cells stimulated with ChIFN-λ. Up-regulated genes induced by ChIFN-λ in both DF1 and LMH cells were considered as chicken type III IFN-stimulated genes (ISGs). **C** Statistics used for the identification of chicken type I, II, and III ISGs in this study.

Compared to the control, 205 up-regulated and 127 down-regulated DEGs were identified in ChIFN-α-stimulated DF1 cells; 299 up-regulated and 221 down-regulated DEGs were identified in ChIFN-γ- stimulated DF1 cells; and 261 up-regulated and 198 down-regulated DEGs were identified in ChIFN-λ- stimulated DF1 cells (Figure 2A, Additional file 4). In LMH cells, we found that 246 DEGs were up-regulated and 310 DEGs were down-regulated after ChIFN-λ stimulation (Figure 2A, Additional file 4). The expression patterns of these DEGs were displayed in heatmaps (Additional file 5), which illustrate the clustering and repeatability of the IFN-treated and normal samples.

Furthermore, GO and KEGG analysis was performed on DEGs in each IFN-treated group (Additional files 6 and 7). ChIFN-α-induced DEGs are mainly enriched in “immune effector process”, “immune system process”, “cytokine production”, “regulation of defense response” for GO terms and “Influenza A”, “Herpes simplex infection”, “RIG-I-like receptor signaling pathway” and “TGF-beta signaling pathway” for KEGG pathways in DF1 cells (Additional files 6A, B). DEGs induced by ChIFN-γ in DF1 cells are mainly enriched in “immune effector process”, “regulation of response to stimulus”, “immune system process”, “cytokine production” for GO terms and “Jak-STAT signaling pathway”, “Cytokine-cytokine receptor interaction”, “Cytosolic DNA-sensing pathway” and “Toll-like receptor signaling pathway” for KEGG pathways (Additional files 6C, D). The DEGs induced by ChIFN-λ in both DF1 and LMH are mainly enriched in “response to other organism”, “response to external biotic stimulus”, “immune effector process”, “immune system process” for GO terms and “Herpes simplex infection”, “Influenza A”, “Jak-STAT signaling pathway” and “RIG-I-like receptor signaling pathway” for KEGG pathways (Additional files 6E–H).

### Identification of ISGs specific for chicken type I, II, III IFNs

All up-regulated DEGs were considered as potential chicken ISGs in this study. Comparisons between up-regulated DEGs induced by ChIFN-λ in DF1 cells and those in LMH cells revealed that 86 up-regulated DEGs were identical in the two cell types (Figure 2B). Up-regulated DEGs induced by ChIFN-α and ChIFN-γ in DF1 cells were considered to be potential chicken type I and type II ISGs, respectively. However, the up-regulated genes induced by ChIFN-λ in DF1 cells and LMH cells were considered to be chicken type III ISGs. Therefore, as shown in Figure 2C, we systematically identified 205 chicken type I ISGs, 299 chicken type II ISGs, and 421 chicken type III ISGs. The details of the ISGs in each group are shown in Additional file 8.

**Figure 3:**
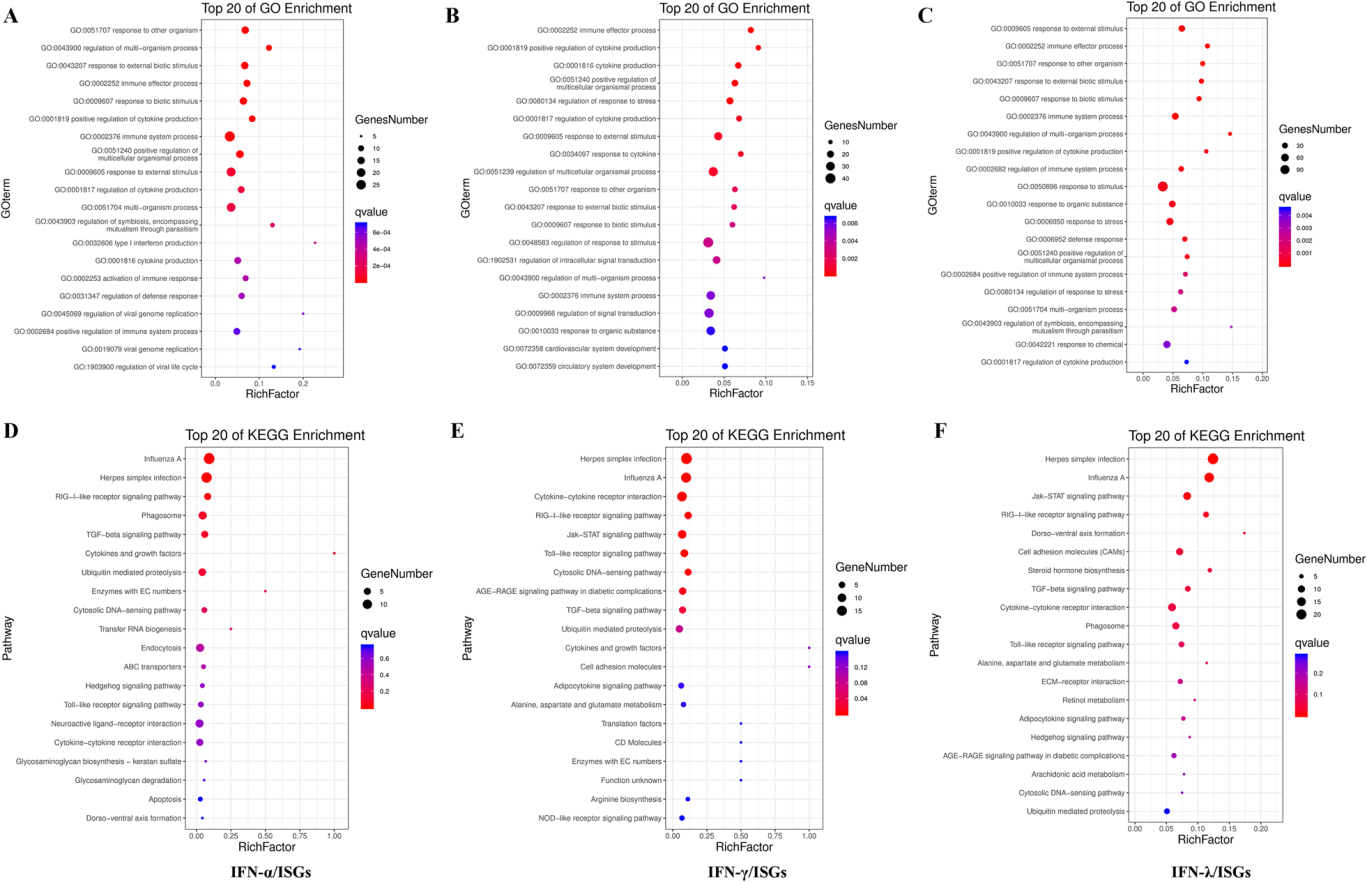
**Enrichment analysis of chicken type I, II and III ISGs.** Top 20 GO biological process terms were selected for type I (A), II (B) and III (C) chicken ISGs enrichment. Top 20 KEGG pathways were selected for type I (D), II (E) and III (F) chicken ISGs enrichment.

Moreover, the three types of chicken ISGs were assigned to various GO categories and KEGG pathways to determine their functional classifications (Figure 3, Additional file 9). In biological process (BP), most of chicken type I ISGs were involved in “immune system process”, “response to external stimulus”, “cytokine production”, “multi-organism process” and “regulation of defense response” (Figure 3A). Chicken type II ISGs were mainly enriched in “immune effector process”, “cytokine production”, “response to external stimulus”, “response to cytokine” and “regulation of multicellular organismal process” (Figure 3B). Chicken type III ISGs were also mainly involved in immune-related BP terms including “response to stimulus”, “immune system process”, “defense response”, “positive regulation of cytokine production” and “immune effector process” (Figure 3C).

For KEGG pathways, the three types ISGs were all mainly enriched in “Influenza A”, “Herpes simplex infection”, “RIG-I-like receptor signaling pathway”, “TGF-beta signaling pathway”, “Cytokine-cytokine receptor interaction” and “Toll-like receptor signaling pathway” (Figures 3D–F).

### RNA‑seq data matched the qPCR data

**Figure 4:**
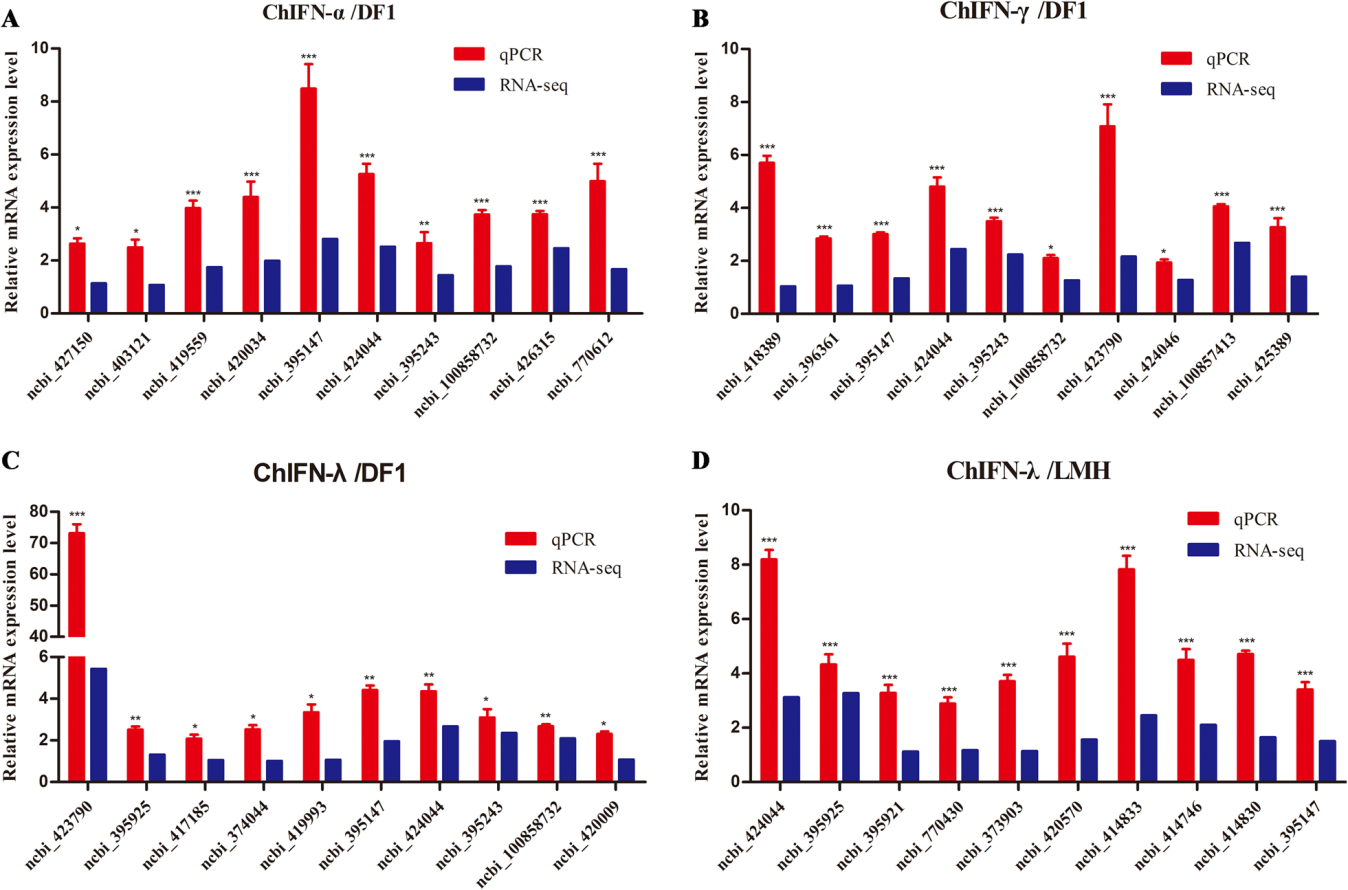
**Validation of RNA-Seq data by qPCR.** ISGs were selected from ChIFN-α-stimulated DF1 cells (**A**); ChIFN-γ- stimulated DF1 cells (**B**); ChIFN-λ- stimulated DF1 cells (**C**); and ChIFN-λ- stimulated LMH cells (**D**). The data of relative mRNA expression level was derived from the ratio of the ChIFN-treatment group results to the control group results. qPCR and RNA-seq results were respectively displayed as 2^−ΔΔCt^ value and the average log_2_ (fold change) values of DEG. Data from qPCR are representative of three independent experiments. *p < 0.05, **p < 0.01, ***p < 0.001. Error bars indicate SEM.

To further evaluate the reliability of the RNA-Seq results, 10 ISGs from each group were randomly selected to validate the relative expression levels in the control and IFN treatment group using qPCR. As shown in Figure 4, the trends in expression of these randomly selected ISGs were consistent with our RNA-seq data (log_2_ (FC)), indicating that the RNA-seq data were reliable.

### Analysis of ISRE and GAS elements in chicken ISGs promoter regions

In mammals, ISRE sequences in promoters of ISGs respond to type I and III IFN signaling, whereas GAS sequences respond to type II IFN signaling [1, 3]. Different types of IFN could induce a unique or partially overlapping set of ISGs [8]. To detect whether the candidate chicken types I, II, and III ISGs contained these specific elements in the promoter region, we further cross-classified these ISGs and searched for ISRE and GAS elements in the promoter regions of the respective genes.

**Figure 5:**
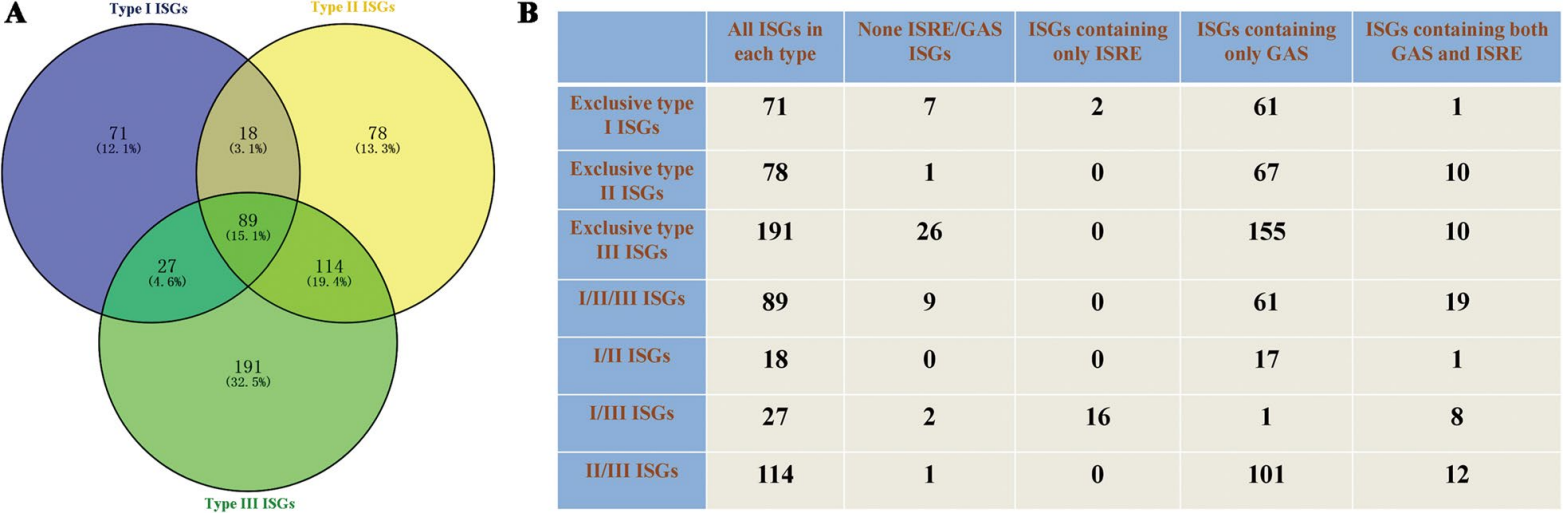
**IFN-stimulated response element (ISRE) and gamma-activated sequence (GAS) elements in the promoter regions of chicken ISGs. A** Venn diagrams of chicken type I, type II, and type III ISGs. **B** ISRE and GAS elements in the promoter of chicken ISGs.

Venn diagrams of the three types of chicken ISGs revealed the existence of 89 ISGs belonging to the intersection of chicken types I, II, and III ISGs; 18 ISGs at the intersection of chicken types I and II ISGs; 27 ISGs at the intersection of chicken types I and III ISGs; and 114 ISGs at the intersection of chicken types II and III ISGs (Figure 5A). Based on these results, we further subdivided the chicken types I, II, and III ISGs into seven categories, including 71 exclusive type I ISGs; 78 exclusive type II ISGs; 191 exclusive type III ISGs; 89 ISGs belonging to types I, II and III ISGs (I/II/III ISGs); 18 ISGs belonging to types I and II ISGs (I/II ISGs); 27 ISGs belonging to types I and III ISGs (I/III ISGs); and 114 ISGs belonging to types II and III ISGs (II/III ISGs) (Figure 5B).

The region at 6 kb upstream of the first exon was selected for searching of ISRE and GAS elements in each ISG group (Additional file 10). Interestingly, we found that the majority of the regulatory elements were GAS elements, even among the exclusive type I and exclusive type III ISGs (Figure 5B and Additional file 10). The exclusive type II ISGs were also found to contain ISREs (Figure 5B and Additional file 10). In addition, no ISRE or GAS elements were found in the promoters of 46 ISGs (Figure 5B). Only two exclusive type I ISGs and 16 type I/III ISGs contained ISREs (Figure 5B).

**Figure 6:**
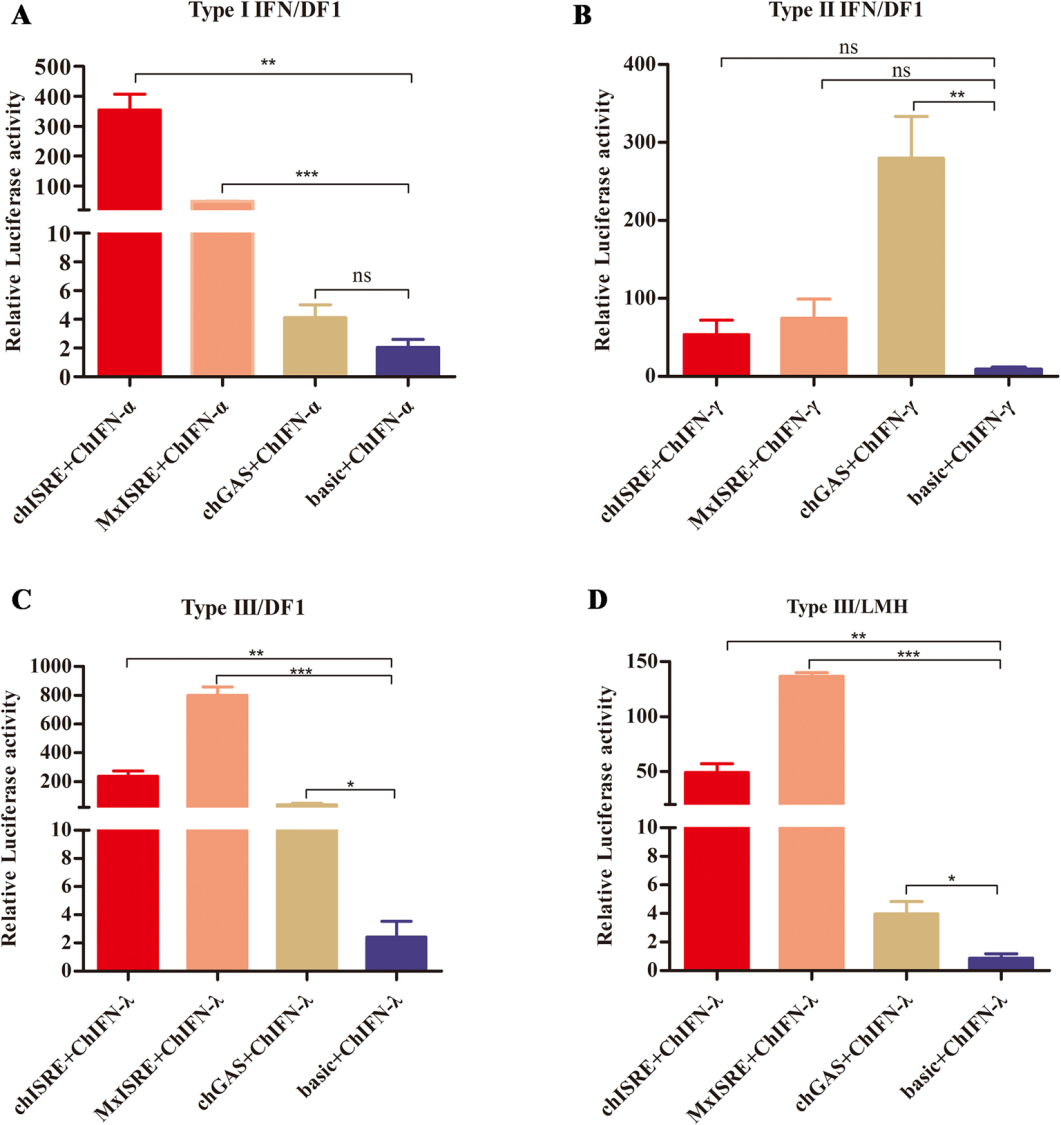
**Activity analysis of IFN-stimulated response element (ISRE) and gamma-activated sequence (GAS) elements after stimulation by chicken IFN. A**–**C** Analysis of activation of ISRE and GAS elements induced by chicken type I, II, and III IFNs in DF1 cells. **D** Analysis of activation of ISRE and GAS elements induced by chicken type III IFN in LMH cells. Data are representative of three independent experiments. ns: not significant (*P* > 0.05), *P < 0.01, **P < 0.01, ***P < 0.001. Error bars indicate SEM.

To further assess whether the ISRE and GAS elements played a role in the transcriptional activation of ISGs after chicken IFN stimulation, luciferase reporter assays were performed in DF1 or LMH cells after transfection with pGL3-MxISRE, pGL3-chISRE, pGL3-chGAS, and pGL3-basic reporter plasmids (in combination with pRL-TK) and IFN-treatment. Figure 6A shows that the presence of ISREs significantly upregulated promoter activation in DF1 cells after ChIFN-α treatment, while this was not observed for GAS elements. As expected, ChIFN-γ significantly activated GAS elements, but not ISRE elements in DF1 cells (Figure 6B). Furthermore, ChIFN-λ treatment was able to significantly activate ISREs in DF1 and LMH cells (Figures 6C, D). Surprisingly, GAS elements were also significantly activated in ChIFN-λ- stimulated DF1 and LMH cells (Figures 6C, D).

## Discussion

Given their key roles in innate immune defense, ISGs have been extensively studied in humans. The identification of type I and II ISGs in the transcriptome of several cell types has been conducted in previous studies [8–12]. Recently, the protein interaction network of ISGs was constructed to extend the landscape of the innate immune system [43]. However, although type III IFN has recently been found to play an important role in the antiviral innate immune response [16, 44, 45], the search for type III ISGs has not yet been carried out systematically. Even more regrettable, the identification of avian ISGs (type T, II and III) is only in its early stage. In our previous study, we systematically identified ISGs induced by chicken IFN-α in chicken peripheral blood mononuclear cells (PBMCs) [25]. Other researchers have systematically studied IFN-α-induced genes in primary chicken embryo fibroblasts or tissues [24, 38]. In view of the imperfect genomic information presently available for chicken, which is still being updated, and the lack of information on types II and III ISGs, we systematically identified chicken types I, II, and III ISGs in the present study.

A forward-looking study previously attempted to compare the expression of genes induced by IFN-β, IFN-γ, and IFN-λ in chickens [46]. Unfortunately, that study only selected two genes, *Mx* and *OAS* [46]. In the present study, 205 type I, 299 type II, and 421 type III ISGs were systematically identified in the chicken. Previous studies have suggested that IFN-λ mainly plays a role in epithelial cells [30]. Thus, epithelial cells were deliberately selected in the present study, in addition to the fibroblasts that are commonly used. Unexpectedly, 261 type III ISGs were identified in DF1 cells, whereas 246 type III ISGs were identified in LMH cells. These results suggest that chIFN-λ has the potential to exert antiviral effects in chicken fibroblasts. In another study, we demonstrated that chIFN-λ could restrict the replication of avian leukosis virus subgroup J (ALV-J) in DF1 cells [47]. However, only 80 type III ISGs in DF1 and LMH cells were common. This result suggests that IFN can induce different ISGs in different types of cells. In addition, chicken cholesterol 25-hydroxylase (*chCH25H*), another chicken ISG, was identified and characterized in our previous research, which showed high expression in PBMCs after 6 h of treatment with ChIFN-α, and in DF1 cells at 24 h and 48 h post chIFN-α treatment [25]. However, in the present study, there was no significant increase in *CH25H* expression after treatment with ChIFN-α for 6 h in DF1 cells. Indeed, even for a particular cell type, some genes were induced by IFN at one time point but not at another time point. The phenomenon of different levels of induction at different time points was also evident in previous studies of ISGs [12]. Therefore, the strategy of identifying chicken ISGs using the present protocol is not exhaustive. In future studies, we aim try to use more cell types, different time points, and different IFN doses to increase the coverage of ISGs.

The prevailing view from studies in mammalian systems is that the complex of ISGF3 (type I and III IFNs) and GAF (type II IFN) translocates to the nucleus and binds to ISRE (type I and III ISGs) and GAS (type II ISGs) elements present in the promoters, thereby initiating the transcription of the respective ISGs [1]. We hypothesized that ISREs would be present in the promoters of all type I and III ISGs. Surprisingly, ISREs were identified only in a minority of chicken ISGs. Furthermore, ISREs were also found in the promoters of some type II ISGs. Moreover, GAS elements were present in most ISGs that included type I, II, and III. Previous studies have reported that ISRE and GAS elements are uncommon in promoters of type I ISGs in chicken [38].

The question can therefore be raised to what extent the expression of chicken ISGs is regulated via binding of specific transcription complexes to ISRE and GAS elements in promoters as is well established for mammalian ISGs. Many of the chicken types I and III ISGs do not contain classical ISREs within their promoters, and details of their transcriptional regulation require further characterization. In fact, the active ISGF3 complex interaction with the core of the ISRE is mediated by IRF9 [48]. However, chickens have no mammalian homologous *IRF9* gene. The currently annotated chicken *IRF9* is in fact an ortholog of *IRF10* [3]. Therefore, does the induction of type I and III ISGs in chickens not require ISRE or IRF9? Does chicken IRF10 play a role in type I and III ISGs production? Canonical and non-canonical pathways that induce the expression of type I and III ISGs in chickens are interesting research directions. Additionally, we found that ChIFN-λ could activate promoters with GAS elements in DF1 and LMH cells. These results suggest that ChIFN-λ has a potentially broader ISG induction profile than chicken types I and II IFN.

The presence or absence of IFN response elements in the promoter region of ISGs may be related not only to the mechanism of initiation of transcription, but also to their biological function. A study found that the antiviral activities of IFNs depended on a set of IFN-sensitive genes (“robust” genes) with canonical IFN response elements (ISRE), whereas these elements were not found in the promoters of ISGs that mediate the anti-proliferative responses of IFNs [49]. This result suggests that when we conduct large-scale antiviral ISGs screening, it may be possible to narrow the screening range according to whether the ISGs promoter contains IFN response elements (ISRE and GAS). Indeed, in our study, we found that some classic antivirus ISGs, such as *MX*, *EIF2AK2* (*PKR*), *RSAD2* (*viperin*), *IFITM3*, *ZC3HAV1*(*ZAP*) (Additional file 10), contain IFN response elements in their promoters.

Current information on chicken ISGs is limited. The profile of ISGs, the mechanism of their production and their antiviral mechanisms are all subject for further studies. Even in mammals, among the hundreds of ISGs identified, few have been characterized for their contributions to antiviral immune responses. Recent efforts have been aimed at identifying which ISGs are antiviral and at further characterizing their mechanisms of action [25, 50, 51]. Our results provide a first attempt for systematic identification of chicken ISGs, which will lay the foundation for further research into their characteristics and antiviral functions.

In summary, type I, II and III IFN ISGs were systematically identified in chicken cells and an initial analysis of response elements in their promoters was carried out. Here, we identified 205 type I, 299 type II, and 421 type III ISGs in the chicken. The antiviral activities of chicken ISGs and their biological functions in vivo will require further investigation.

## Supplementary information

**Additional file 1. Activity detection of recombinant ChIFN-γ and ChIFN-λ against vesicular stomatitis virus (VSV) in vitro.** (A) Cytopathic effects (CPE) caused by VSV (100 TCID_50_) in DF1 cells preincubated with different concentrations of recombinant ChIFN-γ. Dilution factor (only showed the CPE results of two dilution factors at the critical point): 4^−4^ (no CPE), 4^−5^ (CPE appearance). (B) CPE induced by VSV (100 TCID_50_) in DF1 cells preincubated with different concentrations of recombinant ChIFN-λ. Dilution factor 4^−2^ (no CPE), 4^−3^ (CPE appearance). (C) CPE induced by VSV (100 TCID_50_) in LMH cells preincubated with different concentrations of recombinant ChIFN-λ. Dilution factor 4^−3^ (no CPE), 4^−4^ (CPE appearance). NC, negative control (mock treated cells); VSV + , positive control (cells are directly inoculated with VSV without IFN treatment).

**Additional file 2. RNA-Seq data statistics**.

**Additional file 3. Volcano plot of identified DEGs between ChIFN-treated cells and untreated cells.** Volcano plot of DEGs in DF1 cells treated with 1000 UI/mL ChIFN-α (A), ChIFN-γ (B) and ChIFN-λ (C) at 6 h post treatment. (D) Volcano plot of DEGs in LMH cells treated with ChIFN-λ. The red spots represent significantly up-regulated DEGs. The green spots represent significantly down-regulated DEGs. The black spots indicate no significantly differential expression.

**Additional file 4. Properties of identified DEGs**.

**Additional file 5. Heatmap of DEGs in ChIFN-treated samples and their untreated controls**. Heatmap of DEGs in DF1 cells treated with 1000 UI/mL ChIFN-α (A), ChIFN-γ (B) and ChIFN-λ (C) at 6 h post treatment. (D) Heatmap of DEGs in LMH cells treated with ChIFN-λ.

**Additional file 6. Enrichment analysis of DEGs induced by ChIFN treatment in DF1 and LMH cells**. Top 20 GO biological process terms were selected for type I (A), II (C) and III (E) IFN-induced DEGs in DF1 cells and type III IFN-induced DEGs in LMH cells (G). Top 20 KEGG pathways were selected for type I (B), II (D) and III (F) IFN-induced DEGs in DF1 cells and type III IFN-induced DEGs in LMH cells (H).

**Additional file 7. GO and KEGG pathway analysis of DEGs**.

**Additional file 8. Details of chicken types I, II, and III ISGs**.

**Additional file 9. GO and KEGG pathway analysis of chicken ISGs**.

**Additional file 10. IFN-stimulated response elements (ISRE) and gamma-activated sequence (GAS) elements identified in the promoter region of chicken type I, II and III ISGs**.
