# Supplementary figures and images for: Systematic identification of chicken type I, II and III interferon-stimulated genes
Source: Vet Res. 2020 May 24;51:70. doi: 10.1186/s13567-020-00793-x (PMC7245633; doi:10.1186/s13567-020-00793-x)

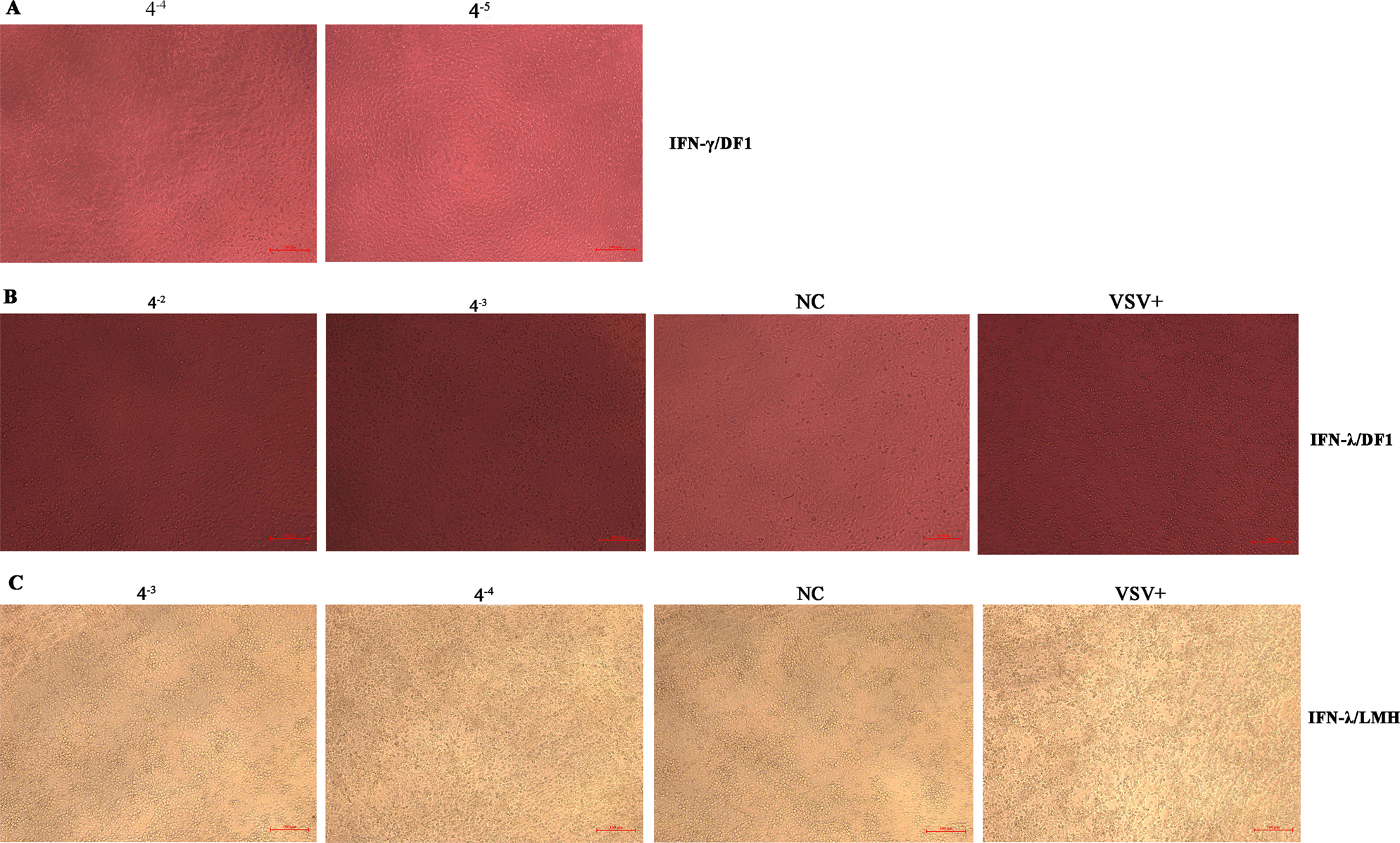

Supplement: Supplementary file 1 — Additional file 1. Activity detection of recombinant ChIFN-γ and ChIFN-λ against vesicular stomatitis virus (VSV) in vitro. (A) Cytopathic effects (CPE) caused by VSV (100 TCID50) in DF1 cells preincubated with different concentrations of recombinant ChIFN-γ. Dilution factor (only showed the CPE results of two dilution factors at the critical point): 4−4 (no CPE), 4−5 (CPE appearance). (B) CPE induced by VSV (100 TCID50) in DF1 cells preincubated with different concentrations of recombinant ChIFN-λ. Dilution factor 4−2 (no CPE), 4−3 (CPE appearance). (C) CPE induced by VSV (100 TCID50) in LMH cells preincubated with different concentrations of recombinant ChIFN-λ. Dilution factor 4−3 (no CPE), 4−4 (CPE appearance). NC, negative control (mock treated cells); VSV + , positive control (cells are directly inoculated with VSV without IFN treatment). [file 13567_2020_793_MOESM1_ESM.tif]

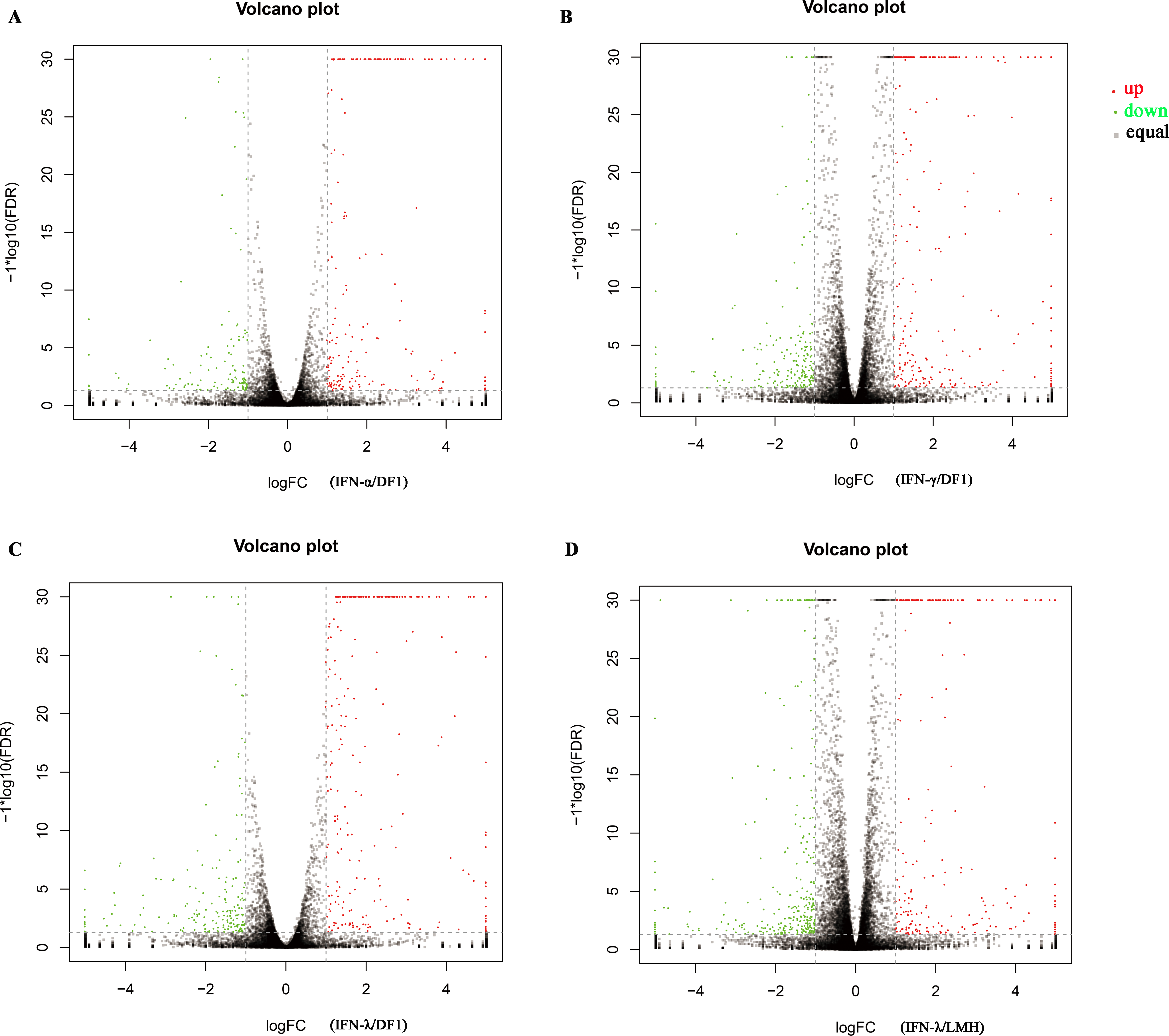

Supplement: Supplementary file 3 — Additional file 3. Volcano plot of identified DEGs between ChIFN-treated cells and untreated cells. Volcano plot of DEGs in DF1 cells treated with 1000 UI/mL ChIFN-α (A), ChIFN-γ (B) and ChIFN-λ (C) at 6 h post treatment. (D) Volcano plot of DEGs in LMH cells treated with ChIFN-λ. The red spots represent significantly up-regulated DEGs. The green spots represent significantly down-regulated DEGs. The black spots indicate no significantly differential expression. [file 13567_2020_793_MOESM3_ESM.tif]

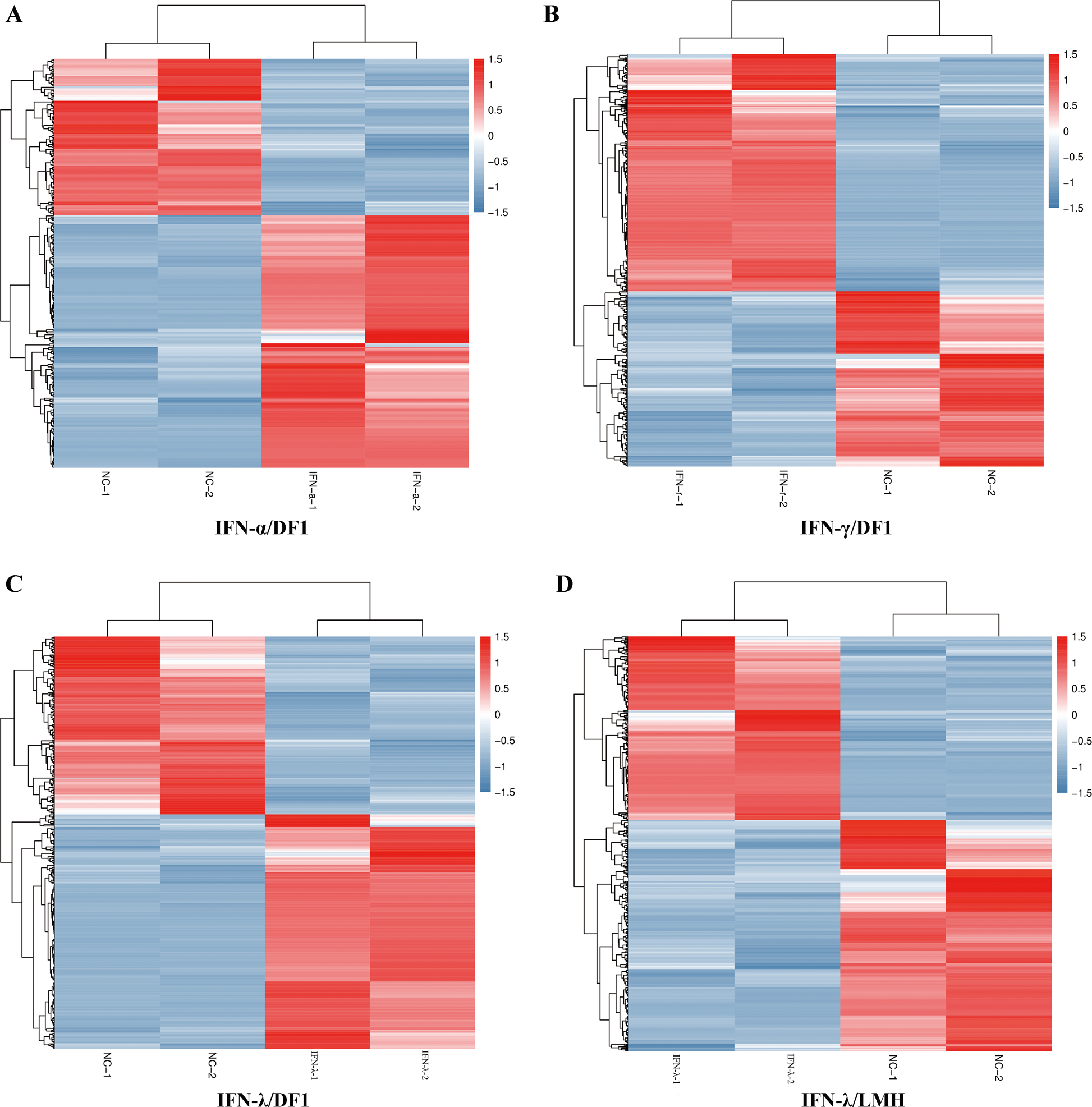

Supplement: Supplementary file 5 — Additional file 5. Heatmap of DEGs in ChIFN-treated samples and their untreated controls. Heatmap of DEGs in DF1 cells treated with 1000 UI/mL ChIFN-α (A), ChIFN-γ (B) and ChIFN-λ (C) at 6 h post treatment. (D) Heatmap of DEGs in LMH cells treated with ChIFN-λ. [file 13567_2020_793_MOESM5_ESM.tif]

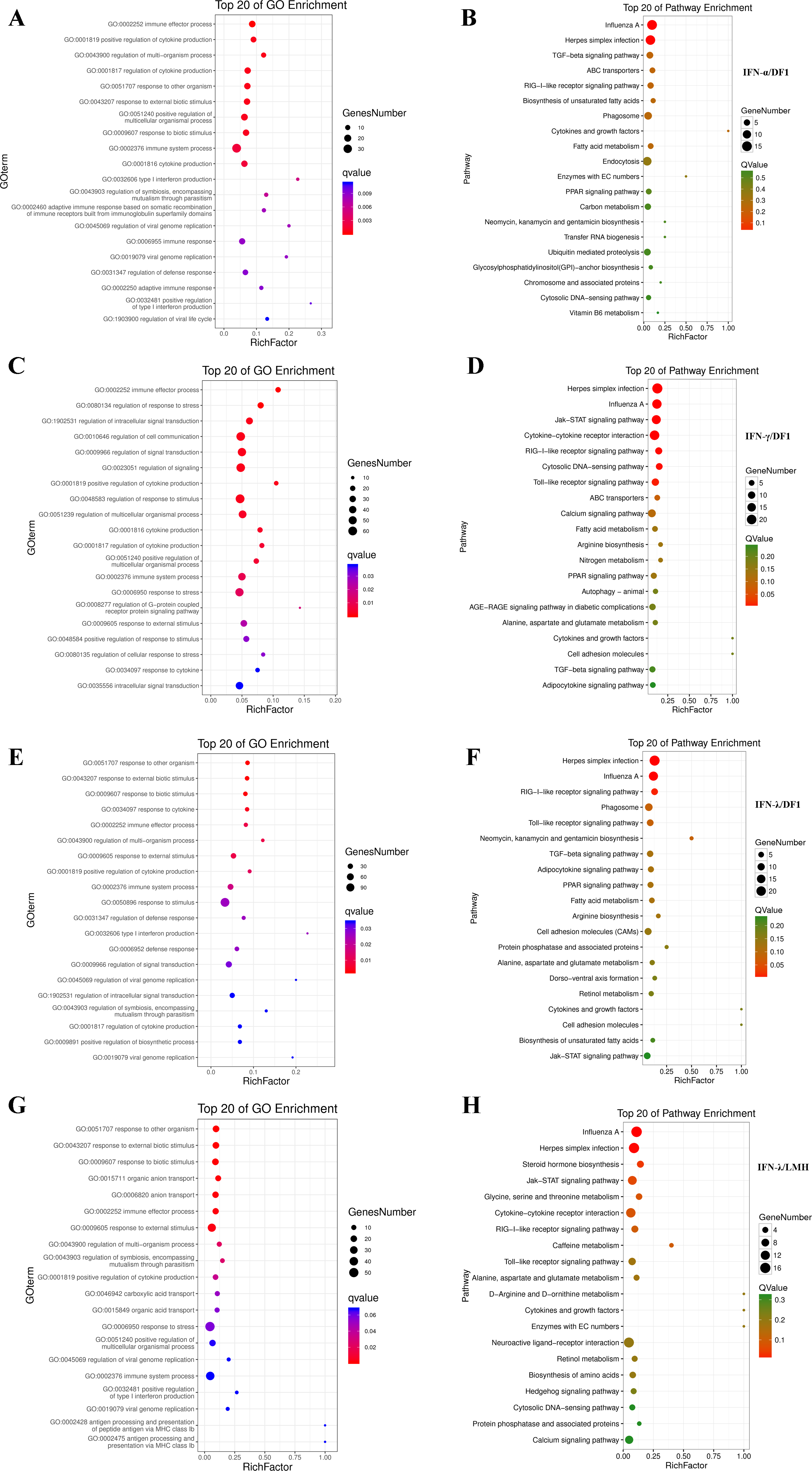

Supplement: Supplementary file 6 — Additional file 6. Enrichment analysis of DEGs induced by ChIFN treatment in DF1 and LMH cells. Top 20 GO biological process terms were selected for type I (A), II (C) and III (E) IFN-induced DEGs in DF1 cells and type III IFN-induced DEGs in LMH cells (G). Top 20 KEGG pathways were selected for type I (B), II (D) and III (F) IFN-induced DEGs in DF1 cells and type III IFN-induced DEGs in LMH cells (H). [file 13567_2020_793_MOESM6_ESM.tif]
