# Supplementary material for: Systematic identification of chicken type I, II and III interferon-stimulated genes
Source: Vet Res. 2020 May 24;51:70. doi: 10.1186/s13567-020-00793-x (PMC7245633; doi:10.1186/s13567-020-00793-x)
Supplement: Supplementary file 2 — Additional file 2. RNA-Seq data statistics. [file 13567_2020_793_MOESM2_ESM.docx]

**Additional file 2 RNA-Seq data statistics**

| **Sample name** | **Raw Data (bp)** | **Clean Data (bp)** | **Clean Reads** | **Unique Mapped Reads** | **Multiple Mapped**  **Reads** | **Mapping Ratio** |
| --- | --- | --- | --- | --- | --- | --- |
| **NC-1(DF1)** | 6012741300 | 5815070665 | 38842234 | 33472629 (86.18%) | 193000 (0.50%) | 86.67% |
| **NC-2(DF1)** | 6313965300 | 6078134474 | 40654034 | 34929285 (85.92%) | 210568 (0.52%) | 86.44% |
| **IFN-α-1(DF1)** | 6072765000 | 5861703678 | 39202474 | 33757700 (86.11%) | 194028 (0.49%) | 86.61% |
| **IFN-α-2(DF1)** | 6335686800 | 6118046126 | 41077536 | 35203345 (85.70%) | 197100 (0.48%) | 86.18% |
| **IFN-γ-1(DF1)** | 6643094400 | 6435035550 | 43006014 | 37250982 (86.62%) | 218138 (0.51%) | 87.13% |
| **IFN-γ-2(DF1)** | 5436626700 | 5264941204 | 35243414 | 30585527 (86.78%) | 174186 (0.49%) | 87.28% |
| **IFN-λ-1(DF1)** | 6246674100 | 6057039092 | 40513094 | 35068219 (86.56%) | 199380 (0.49%) | 87.05% |
| **IFN-λ-2(DF1)** | 6031362900 | 5802702911 | 39183944 | 33643523 (85.86%) | 195288 (0.50%) | 86.36% |
| **NC-1(LMH)** | 6347541000 | 6135378317 | 41300448 | 35345774 (85.58%) | 222018 (0.54%) | 86.12% |
| **NC-2(LMH)** | 7117463100 | 6875068594 | 45318556 | 38038398 (83.94%) | 234024 (0.52%) | 84.45% |
| **IFN-λ-1(LMH)** | 7280218500 | 7022005236 | 47180760 | 40059767 (84.91%) | 272168 (0.58%) | 85.48% |
| **IFN-λ-2(LMH)** | 5711515200 | 5505772616 | 36741888 | 31157774 (84.80%) | 216156 (0.59%) | 85.39% |
